# The effect of the necrotic area on the biomechanics of the femoral head - a finite element study

**DOI:** 10.1186/s12891-020-03242-0

**Published:** 2020-04-06

**Authors:** Pengfei Wen, Yumin Zhang, Linjie Hao, Ju’an Yue, Jun Wang, Tao Wang, Wei Song, Wanshou Guo, Tao Ma

**Affiliations:** 1 Department of Joint Surgery, Honghui Hospital, Xi’an Jiaotong University, No. 555 Youyi East Road, Xi’an, 710054 Shaanxi China; 2 Department of Orthopedics, Aviation General Hospital, No. 3 Anwaibeiyuan Road, Chaoyang District, Beijing, 100020 China; 3 Center for Osteonecrosis and Joint Preserving & Reconstruction, Department of Orthopaedic Surgery, Beijing Key Laboratory of Arthritic and Rheumatic Diseases, China-Japan Friendship Hospital, No. 2 Yinghuadong Road, Chaoyang District, Beijing, 100029 China

**Keywords:** Osteonecrosis of the femoral head, China-Japan friendship hospital classification, Finite element analysis, Collapse

## Abstract

**Background:**

Femoral head collapse is the key to the progress of osteonecrosis of the femoral head (ONFH), but the causes of collapse are not completely clear. The better understanding of the progress of femoral head collapse will guide the treatment strategy for ONFH patients. The purpose of this study was to evaluate the biomechanical influence of necrosis area on the collapse of the femoral head by finite element analysis.

**Methods:**

CT and MRI data from the hip joint of a healthy volunteer were collected to establish a finite element (FE) model of a normal hip. Subsequently, five categories of osteonecrosis FE models were established by using the normal model and computer software according to China-Japan Friendship Hospital (CJFH) classification for ONFH. The CJFH system includes five types based on the size and location of necrosis lesions in the femoral head (type M, C, L1, L2, and L3) and the stage of ONFH. The collapse indices of each model were analyzed by FE method, including the displacement, peak von Mises stress and stress index of the simulated necrotic area as well as the lateral pillar contact area of the femoral head to acetabular.

**Results:**

(1) The displacement increments in the simulated necrotic areas of type M, C, L1, L2, and L3 models were 3.75 μm, 8.24 μm, 8.47 μm, 18.42 μm, and 20.44 μm respectively; the peak von Mises stress decrements were 1.50 MPa, 3.74 MPa, 3.73 MPa, 4.91 MPa, and 4.92 MPa respectively; and the stress indices were 0.04, 0.08, 0.08, 0.27, and 0.27 respectively. (2) The displacement increments in the lateral pillar contact areas of five type models were significantly different (*P* < 0.001) and increased in sequence as follows: 1.93 ± 0.15 μm, 5.74 ± 0.92 μm, 5.84 ± 1.42 μm, 14.50 ± 3.00 μm, and 16.43 ± 3.05 μm. The peak von Mises stress decrements were also significantly different (*P* < 0.001) and increased in sequence as follows: 0.52 ± 0.30 MPa, 0.55 ± 0.12 MPa, 0.67 ± 0.33 MPa, 4.17 ± 0.59 MPa, and 4.19 ± 0.60 MPa. (3) The collapse indices including the displacement increments and peak von Mises stress decrements of type L2 and L3 models were markedly higher than those of type M, C, and L1 models (*P* < 0.001).

**Conclusions:**

The collapse indices of the femoral heads of type L2 and L3 FE models were significantly higher than those of type M, C, and L1. Different areas of necrosis result in varied impact on the femoral head collapse.

## Background

Osteonecrosis of the femoral head (ONFH) is a common orthopedic disease, which affects young and middle-aged patients originated from traumatic or non-traumatic issues. Without early intervention and appropriate treatment, up to 80% of ONFH cases eventually turn into femoral head collapse within 1 to 5 years [1–3]. Femoral head collapse is the most significant pathogenic complication of ONFH that requires total hip replacement eventually. These have motivated the recent studies to focus on the mechanism of the femoral head collapse in ONFH patients. Traditional biomechanical analysis and finite element (FE) method were used to study the causes of femoral head collapse [4–6]. It is believed that the collapse of necrotic area of femoral head is directly related to biomechanical factors [7–9], which is mainly due to some reasons: the decrease of stress in necrotic area, the concentration of stress around necrotic bone, the lower elastic modulus and yield strength of bone tissue in ischemic necrotic area compared to normal tissue and the loss of normal mechanical support [6, 10]. Previous studies showed that the location and lesion size of ONFH were major factors of femoral head collapse [4, 11–13]. However, there are some shortcomings in their research, such as the FE model design is relatively simple, only one of the factors is analyzed, and the classification is not considered [6, 14, 15].

The classification of China-Japan Friendship Hospital (CJFH) is based on the size and location of necrosis lesions in the femoral head as well as the stage of ONFH [12, 16]. The CJFH system could accurately predict the occurrence of the femoral head collapse, which plays a guiding role in the hip-preserving strategy and clinical management of early-stage ONFH [17–21]. However, studies found that, when necrosis lesions occurred in the weight-bearing area of the femoral head, the treatment usually did not work well and the rate of femoral head collapse was as high as 94.4–100% [17–19]. Therefore, the causes of femoral head collapse remain to be studied. In this study, five FE models of ONFH were constructed based on CT and MRI data, and their collapse indices were analyzed by FE method. We successfully used the necrotic areas of the femoral head designed that based on the CJFH classification to study the causes of the femoral head collapse. This result brings a better understanding of the influence of the necrosis area on femoral head collapse.

## Methods

**Fig. 1:**
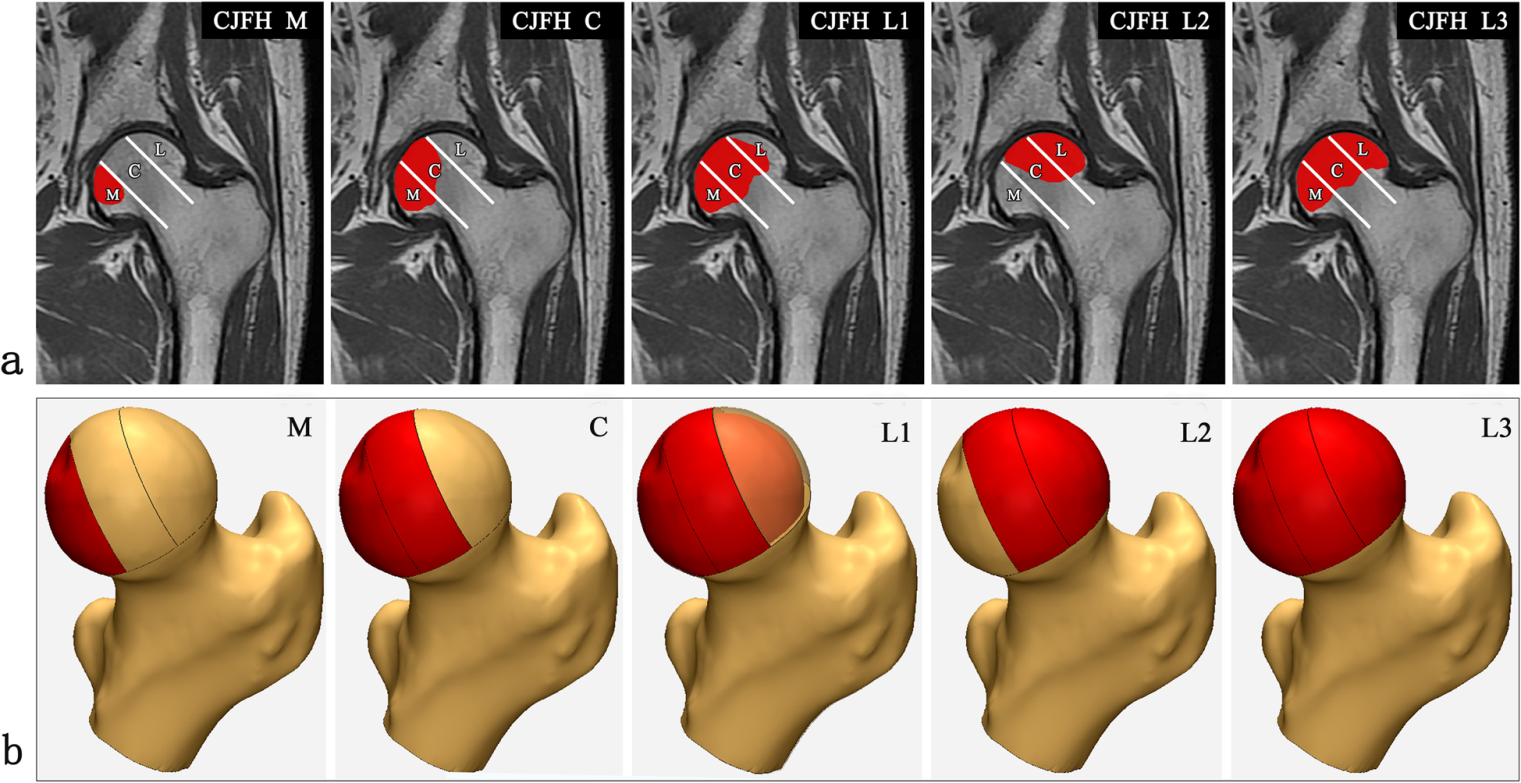
Schematic diagram of China-Japan Friendship Hospital (CJFH) classification for osteonecrosis of the femoral head (ONFH). **a** MRI of CJFH classification for ONFH based on three pillars structure. Type M: the necrosis involved the medial pillar. Type C: the necrosis involved both medial and central pillars. Type L1: the necrosis involved the three pillars but the partial lateral pillar was preserved. Type L2: the necrosis involved the whole lateral pillar and partial central pillar. Type L3: the necrosis involved the three pillars including the cortical bone and marrow. **b** Five three-dimensional CJFH classification models of ONFH. The necrotic area of the femoral head was shown in red

This study was approved by the Ethics Committee of our hospital (No.201902023), and the written informed consent was obtained from the volunteer. The model used in the study was built using ABAQUS 6.14 FE analysis software (Dassault Systems Simulia Corp., Providence, Ri, USA). Details of the model’s construction method, material properties, validation, and convergency testing have been reported in the previous article [22]. The schematic diagram and magnetic resonance imaging (MRI) of CJFH classification for ONFH based on three pillars were shown in Fig. 1a [12, 17]. The different types of CJFH classification are the necrosis lesion involved the medial pillar (Type M), both medial and central pillars (Type C), three pillars but the partial lateral pillar was preserved (Type L1), the whole lateral pillar and partial central pillar (Type L2), three pillars including the cortical bone and marrow (Type L3). Five three-dimensional (3D) CJFH classification models of ONFH were established by the Mimics 17.0 (Materialise Ltd., Leuven, Belgium) and shown in Fig. 1b. The necrotic area of the femoral head was illustrated in red.

Cortical bone, cancellous bone, cartilage, and necrotic area were considered to be isotropic linear elastic materials. The material parameters in each structure were taken from following previous studies (Table 1) [23–26]. The mesh division of the bone structure of the hip joint was established using 10-node modified tetrahedron unit, and where cartilage was established using the hexahedron unit. Finite slip surface contact was applied between the acetabular cartilage and femoral head cartilage (Coulomb friction, with a coefficient of friction of 0.01) [7], and binding constraints were used between the remaining structures.

**Table 1:** Material properties incorporated into the finite element models

Type of tissues	Young’s modulus (MPa)	Poisson’s ratio
Acetabular bone (cortical/cancellous)	17,000/70	0.30/0.20
Femur (cortical/cancellous)	15,100/445	0.30/0.22
Cartilage bone	10.5	0.45
Early necrotic lesion	332.9	0.30

For loading conditions, single leg support (mid-stance phase), corresponded to 30% of the gait cycle as reported previously, was applied [23]. A compressive axial load of 570 N, which accounts for 5/6 of 70 kg body weight, was applied to the nodes of proximal acetabular bone [18, 20]. The muscle contractile forces around the proximal femur were not considered. For boundary conditions, constraints were applied to the displacements of nodes on the symphysis pubis in X- and Y-direction. Nodes on the distal end of the femur were completely fixed to prevent any translation and rotation [19].

Finite element analysis for each model was performed using the software ABAQUS. Simulation data were collected to investigate the collapse indices of the necrosis area as well as the lateral pillar contact area in each model. Collapse indices were selected from previous studies, including displacement variation, peak von Mises stress variation, and stress index. Yang set the yield stress of the necrotic tissue as 5.5 MPa and used the stress index (effective stress/yield strength) to judge the collapse of the femoral head [14]. Fang demonstrated that the critical stress of collapse of necrotic tissue was 0.55 MPa as the criterion of stress index was 0.1 [27]. Additionally, displacement increment was the change of vertical distance at a certain point before and after osteonecrosis and was used to evaluate the degree of femoral head collapse [28]. In other words, the femoral head collapse will occur when the peak von Mises stress, stress index and displacement increment of necrotic bone tissue in the femoral head are greater than these standards.

**Fig. 2:**
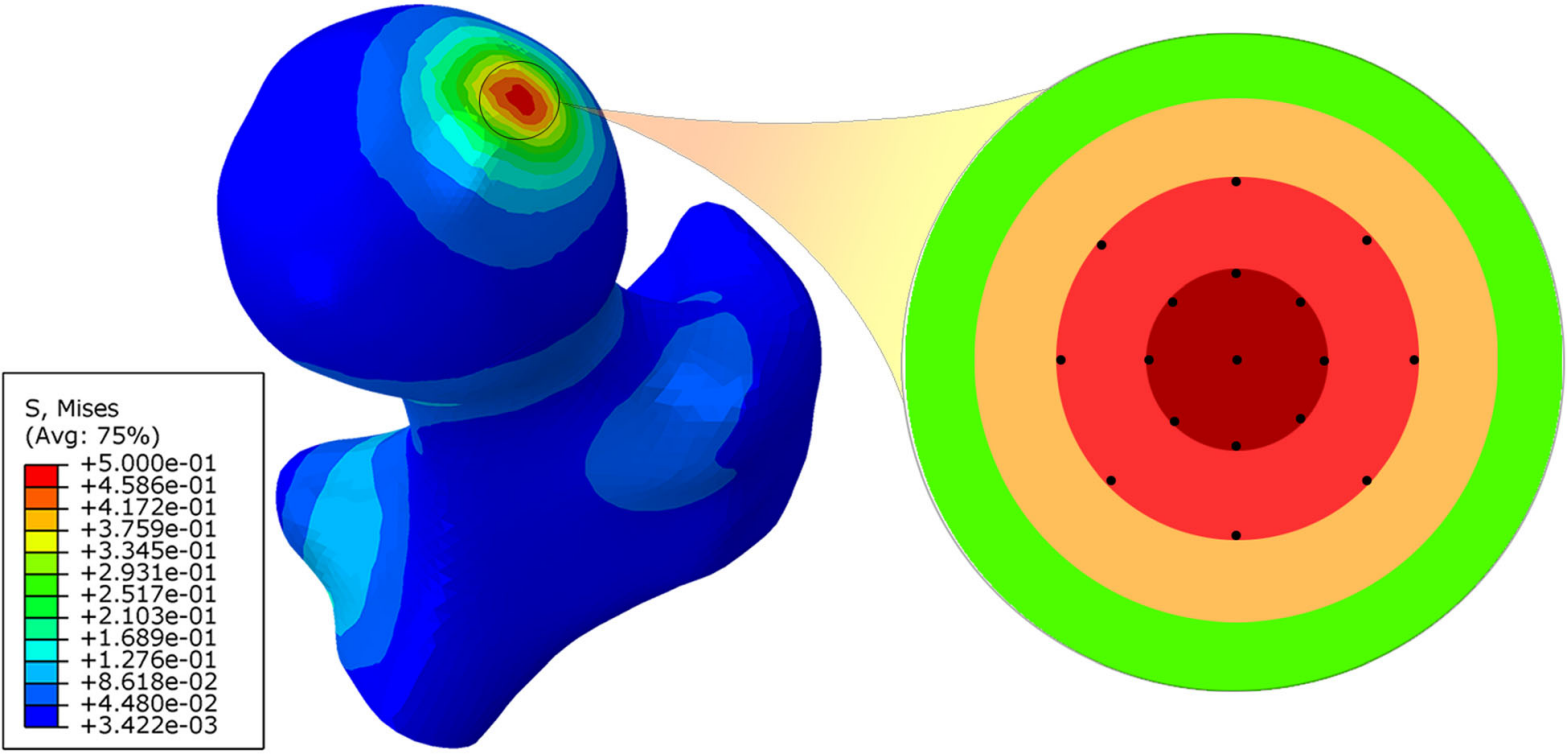
Interested points of the contact area in the lateral pillar of the femoral head. There were 17 black dots distributed around the center of the circle in the figure, and the radius difference of each circle was 0.20 cm

The weight-bearing area of the femoral head is mainly located on the anterolateral side of the femoral head, and the contact area of the femoral head to acetabular was approximately to be circular [22, 23]. Therefore, the lateral contact area of the femoral head was emphatically studied, and the data from the same 17 points on the surface of this area in each model were collected for statistical analysis, as shown in Fig. 2. Quantitative variables were shown as the mean ± standard deviation (^−^*x* ± *s*). ANOVA analysis was applied to analyze the statistical differences among the collapse indices of the five models. As Levene’s variance homogeneity test showed that the variances of the displacement increment and peak von Mises stress decrement of each model were uneven, the Welch variance analysis method was applied to test whether there was any difference among the five models’ data. Then Games Howell test was further applied for post hoc pairwise analysis because of the indeed statistical difference among the five models’ data. Statistical analyses were performed with SPSS 22.0 (SPSS Inc., Chicago, IL, USA). For all tests, *P* < 0.05 was deemed to be significant.

## Results

Distribution of stress and displacement in CJFH classification models.

**Fig. 3:**
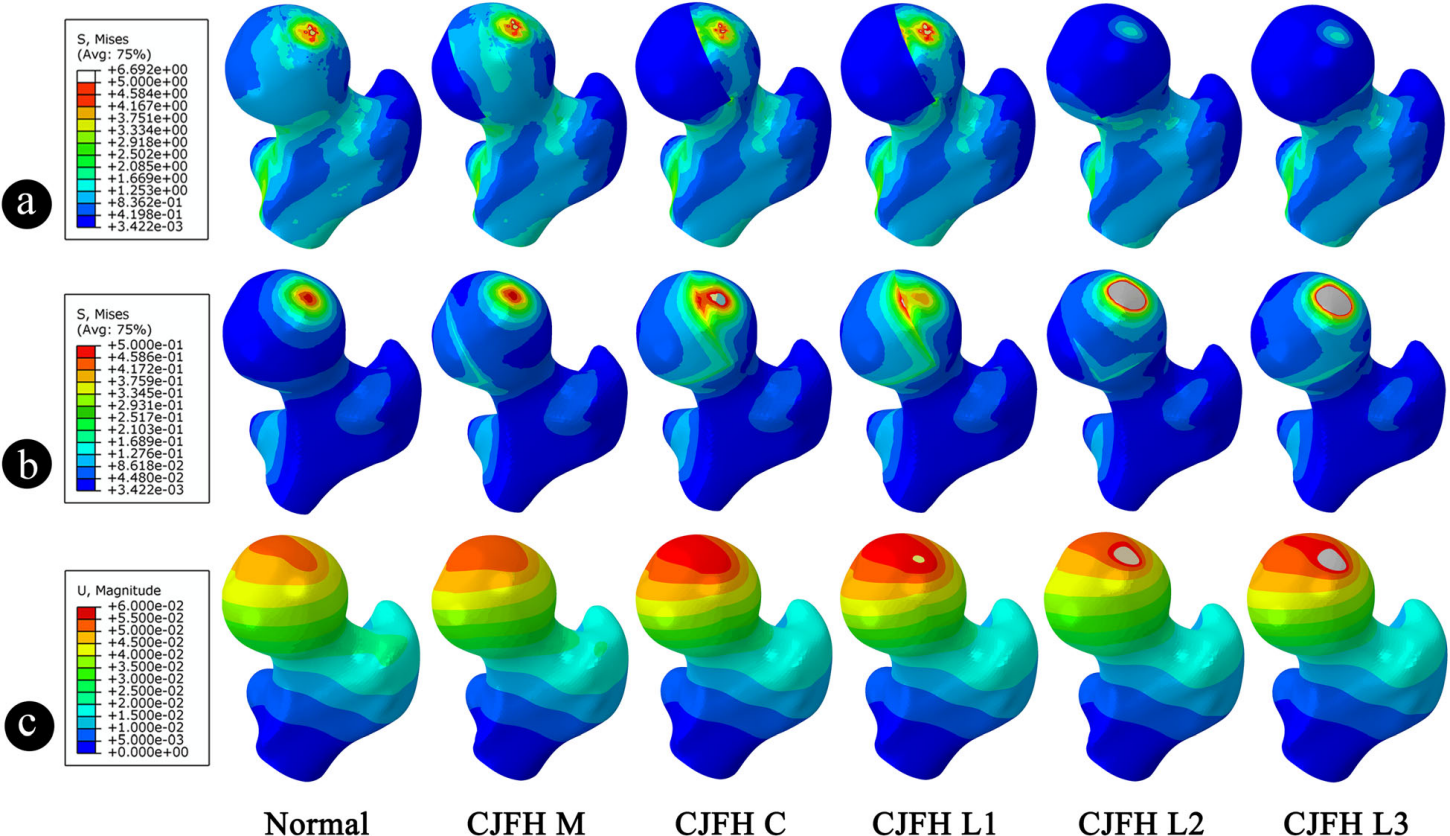
Von Mises stress distribution and displacement nephogram of the CJFH classification models of ONFH. **a** The von Mises stress nephogram of the cortical bone of each model. **b** The von Mises stress nephogram of the cancellous bone of each model. **c** The displacement distribution nephogram of each model. The red areas represent the stress intensive area, while the gray areas represent the area where the stresses have saturated

Figure 3 showed the stress distribution and displacement nephogram of all models. In Fig. 3a and b, in order to obtain a satisfactory stress distribution image for comparative observation, uniform maximum display stress was set to 0.5 MPa and 0.05 MPa, respectively. In Fig. 3a, the peak von Mises stress of the cortical bone was obviously decreased in the lateral pillar of the femoral head of type L2 and L3 models. In contrast, the peak von Mises stress of the cancellous bone was generally increased as shown in Fig. 3b, and the peak displacement of type L2 and L3 models was also noticeably higher than normal (Fig. 3c).

Variation of the collapse indices in the necrotic area of the femoral head in each model.

**Fig. 4:**
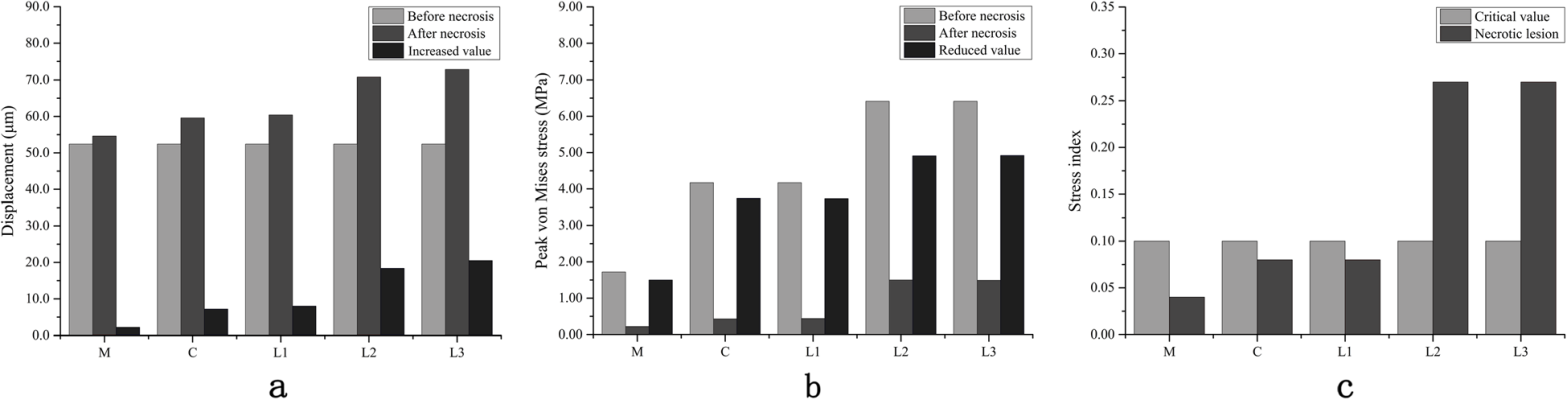
The bar diagrams of the **a** displacement, **b** peak von Mises stress, and (c) stress index of the necrotic area in the models

Table 2 showed the simulation data collected from FE analysis based on the five CJFH type FE models. For type L2 and L3 models, the displacement increments of the necrosis areas were 18.42 μm and 20.44 μm, the peak von Mises stresses were 1.50 MPa and 1.49 MPa, respectively, and the stress indices were both 0.27. Figure 4a showed the displacement variation of the necrosis area increased in the order of type M, C, L1, L2, and L3. The decrement of the peak von Mises stress in the necrosis area of the femoral head also increased in the order of type M, C, L1, L2, and L3, as presented in Fig. 4b. In addition, the stress indices of the femoral necrosis area of type L2 and L3 models were obviously higher than the critical indicator of 0.10 (Fig. 4c). These showed that the collapse indices of the necrosis areas of type L2 and L3 models were clearly higher than those of the other models.

**Table 2:** The displacement, peak von Mises stress, and stress index of the necrotic area in the models

Models	Displacement (μm)			Peak von Mises stress (MPa)			Stress index of necrotic area*
	Nomal	Necrotic area	Increment	Nomal	Necrotic area	Decrement	
M	48.02	51.77	3.75	1.72	0.22	1.50	0.04
C	51.11	59.34	8.24	4.17	0.43	3.74	0.08
L1	51.11	59.58	8.47	4.17	0.44	3.73	0.08
L2	52.42	70.84	18.42	6.41	1.50	4.91	0.27
L3	52.42	72.86	20.44	6.41	1.49	4.92	0.27

*Stress index = effective stress/yield strength. Microfractures form in necrotic lesions when the stress index is > 0.1 and the peak stress is higher than the critical stress (0.55 MPa). Type M: the necrosis involved the medial pillar. Type C: the necrosis involved both medial and central pillars. Type L1: the necrosis involved the three pillars but the partial lateral pillar was preserved. Type L2: the necrosis involved the whole lateral pillar and partial central pillar. Type L3: the necrosis involved the three pillars including the cortical bone and marrow

Variation of the collapse indices in the contact area of the lateral pillar of the femoral head in each model.

**Fig. 5:**
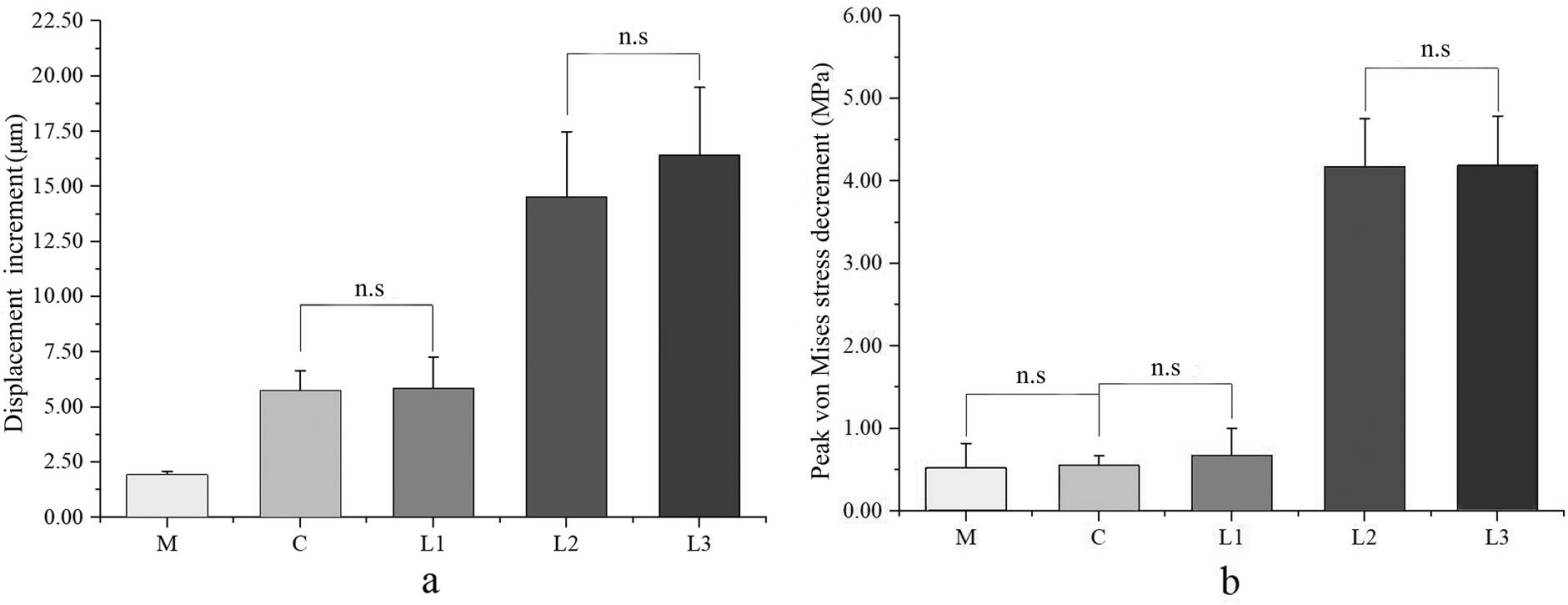
The bar diagrams of **a** displacement increment and **b** peak von Mises stress decrement of the lateral pillar contact area in the models. n.s represents *P*>0.05. The differences between the other unmarked pairs in the figure were statistically significant (*P* < 0.001)

Table 3 showed the displacement changes and stress decrements of the lateral pillar contact area in the five models. Firstly, there were significant differences of the displacement changes among the five models, F (432.630) = 252.469, *P* < 0.001. As shown in Fig. 5a, the displacement changes of models increased in the order of type M, C, L1, L2 to L3, where no significant difference was observed between C type and L1 type models as well as between L2 type and L3 type models (*P* = 0.999, *P* = 0.359, respectively). Secondly, there were significant differences of stress decrements among the five models, F (435.961) = 283.223, *P* < 0.001. The peak von Mises stress decrements of all models increased in the order of type M, C, L1, L2 to L3, as showed in Fig. 5b. It has to be noted that there was no significant difference between type M and C, C and L1, M and L1, and L2 and L3 (*P* = 0.997, *P* = 0.641, *P* = 0.671, *P* = 1.000, respectively). Finally, the displacement changes and peak von Mises stress decrements in type L2 and L3 models were significantly higher than the others (*P* < 0.05).

**Table 3:** The displacement increment and von Mises stress decrement of the lateral pillar contact area in the models

	M (^−^*x* ± *s*)	C(^−^*x* ± *s*)	L1(^−^*x* ± *s*)	L2(^−^*x* ± *s*)	L3(^−^*x* ± *s*)	*P* value
Displacement increment (μm)	1.93 ± 0.15	5.74 ± 0.92	5.84 ± 1.42	14.50 ± 3.00	16.43 ± 3.05	< 0.001
Von Mises stress decrement (MPa)	0.52 ± 0.30	0.55 ± 0.12	0.67 ± 0.33	4.17 ± 0.59	4.19 ± 0.60	< 0.001

Type M: the necrosis involved the medial pillar. Type C: the necrosis involved both medial and central pillars. Type L1: the necrosis involved the three pillars but the partial lateral pillar was preserved. Type L2: the necrosis involved the whole lateral pillar and partial central pillar. Type L3: the necrosis involved the three pillars including the cortical bone and marrow

## Discussion

Bone trabecular microfracture appears in the early stage of ONFH and gradually develops into subchondral bone fracture, which eventually leads to the collapse of the femoral head. The aggravation of hip pain and the decline of joint function would seriously affect the quality of the patient’s life as the patient’s condition aggravates. Some studies have expounded the femoral head collapse from biological factors or mechanical factors. Yang pointed out that biomechanical base of the femoral head collapse was a combined result of poor function condition of internal structure of osteonecrosis, the elastic modulus of the necrotic bone, and the yield strength [14]. However, many studies have reported that type L2 and L3 ONFH patients had poor prognoses and treatment effects in combination with numerous clinical follow-up data, up until now, the causes of collapse have not been completely understood. The purpose of this study was to evaluate the influence of necrosis area on femoral head collapse and disease progression of ONFH, which could provide a better understanding for the poor prognosis and treatment effects in L2 and L3 ONFH patients.

In this study, the simulated necrotic areas of type L2 and L3 FE models were considered to have a higher risk of collapse than those of other types. Bone structures were more prone to occur local microfractures when the peak von Mises stresses exceed their normal bearing capacity. At the same time, bone structural remodeling happened to compensate for local bone loss. If there were too many micro-fractures that happened to the same area, the femoral head would collapse in the end. Yang pointed out that the yield stress of necrotic bone is 5.5 MPa, the stress index is 0.1, and the critical stress of collapse is 0.55 MPa [14]. In this study, the peak von Mises stresses of the necrosis areas in type M, C, and L1 ONFH models were 0.22 MPa, 0.43 MPa, and 0.44 MPa, respectively, which did not exceed the critical stress. Simultaneously, the stress indices of those areas were also lower than the critical value. Therefore, the risk of collapse in the necrosis areas of these three models was considered to be low. In contrast, the peak von Mises stresses and stress indices of the necrotic areas in type L2 and L3 ONFH models were notably higher than the critical value, which leads to significantly increased collapse risk of the necrotic area in these two types.

The lateral pillar areas of the femoral head, contacting with acetabular, of type L2 and L3 FE models were more prone to collapse than those of other models. Some studies revealed that the collapse areas of the femoral head in patients with ONFH are mainly located in the anterolateral femoral head, which provides possible reasons for the collapse as the anterolateral area is the main load-bearing area of the femoral head [23, 29, 30]. Therefore, this study focused on the analysis of the load-bearing area of the lateral pillar of each model. This study found the displacement increments and the peak von Mises stress decrements of those areas in type L2 and L3 models (equal to the necrotic area in these two models) were significantly greater than those in the other three type models. This indicated that the higher risk of femoral head collapse and worse mechanical load-bearing capacity increased significantly in type L2 and L3 ONFH patients.

The cortical areas of the lateral pillar of the femoral head in type M and C ONFH patients all preserved, and the lateral pillar cortex bears the main down-load. As a result, the displacement and peak von Mises stress changes of the lateral pillar contact areas of the femoral head in these two models are not significant. Why are the collapse indices of the femoral head of type L1 FE model lower than those of type L2 and L3? Brown TD [9] and Guo [31] pointed out that the cortical bone of the femoral head played a very important role in stress-bearing. Since the elastic modulus of cortical bone is much larger than that of cancellous bone, the cortical bone of the femoral head bears most of the load. Meanwhile, cancellous bone bears less load than cortical bone due to the stress shielding effect. Compared with type L2 and L3 models of ONFH, the cortical bone of the L1 model remained intact, so the changes of the displacement and peak von Mises stress were relatively small. To sum up, the displacement increments and peak von Mises stress decrements of the lateral pillar contact areas in the type M, C, and L1 ONFH models were relatively small due to the well-preserved lateral pillar cortex. However, when cortical osteonecrosis involved in the lateral pillar, the changes of displacement and peak von Mises stress of the lateral pillar contact area could be significantly higher.

At present time, there are various methods for the treatment of early-stage ONFH, such as extracorporeal high-energy shock wave therapy [17], core decompression [32], tantalum rod implantation [7], silk protein rod implantation [5], bone grafting [18], and rotational osteotomy [33]. Reconstruction of mechanical support of the femoral head is the key to the treatment of ONFH. This study found that the overall distribution of mechanical transmission of the hip joint remained practically unchanged in patients with well-preserved cortical bone in the lateral pillar of the femoral head. Therefore, a good therapeutic effect can be achieved by hip preservation therapy together with well mechanical support reconstruction for patients with type M, C, and L1 ONFH. However, as normal mechanical conduction of the femoral head was severely impaired in patients with type L2 and L3 ONFH, treatments such as extracorporeal shock wave, bone grafting, and tantalum implantation were hard to reconstruct mechanical support and had poor results. Although rotational femoral head osteotomy may reconstruct the mechanical support of the lateral pillar in L2 type ONFH patients, but it is not effectual in L3 type ONFH patients. These are consistent with some previous researches [12, 17–19]. This study just explained the cause of the femoral head collapse and treatment effect from the biomechanical point of view. Femoral head collapse may also be the result of impairment of blood supply caused by factors such as corticosteroid use, alcoholism, hypercoagulation, vascular endothelial dysfunction [2, 32, 34]. Meanwhile, the therapeutic effect was closely related to the treatment strategy, the stage and classification of ONFH, and the pathogenic factors [11, 18, 32].

One limitation of this study could be that the structure of the FE models was specific to the volunteer’s formation as constructed from the data of computerized tomography (CT) and magnetic resonance imaging (MRI) images. The structure of the hip joint was simplified for FE analysis, and ligaments, capsules, and musculatures of the hip joint were not taken into consideration. Another limitation could be that the ideal 3D ONFH models would hardly reflect all the details of the complicated necrotic tissue structure of patients in clinical perspective. Other individual ONFH models could be tested as a future direction. Finally, the material property of the bone structure was considered as linear elastic and homogeneous for simplification, as the cortical and cancellous bones contain spatial inhomogeneity in their properties.

## Conclusions

In summary, this study designed five CJFH classification ONFH models and then used FE analysis method to analyze the biomechanical changes of these models under compressive axial load. It was found that the displacement increments, peak von Mises stress decrements and stress indices of L2 and L3 type ONFH models were significantly higher than those of M, C, and L1 type models in the lateral contact areas or in the necrosis areas. These may explain, in the view of biomechanical causes, the poor treatment effect and prognosis for both L2 and l3 type ONFH patients.

## Data Availability

The datasets used and analyzed during the current study are available from the corresponding author upon reasonable request.

## Abbreviations

ONFH: Osteonecrosis of the femoral head
CJFH: China-Japan Friendship Hospital classification
CT: Computerized Tomography
MRI: Magnetic resonance imaging
FE: Finite element
3D: Three-dimensional
